# Supplementary material for: Honeycomb Biosilica in Sponges: From Understanding Principles of Unique Hierarchical Organization to Assessing Biomimetic Potential
Source: Biomimetics (Basel). 2023 Jun 3;8(2):234. doi: 10.3390/biomimetics8020234 (PMC10296127; doi:10.3390/biomimetics8020234)
Supplement: Supplementary file 1 [file biomimetics-08-00234-s001.zip › biomimetics-2415788-supplementary.pdf]

Article

Honeycomb Biosilica in Sponges: from Understanding Principles of Unique Hierarchical Organization to Assessing Biomimetic Potential

AlonaVoronkina[1,2], Eliza Romanczuk-Ruszuk[3], Robert E. Przekop[4], Pawel Lipowicz[3], Ewa Gabriel[4,5], KorbinianHeimler[6], Anika Rogoll[6], Carla Vogt[6], Milosz Frydrych[5], Pawel Wienclaw[7], Allison L. Stelling[8], Konstantin Tabachnick[9], Dmitry Tsurkan[2] and Hermann Ehrlich[4,*]

[1] Pharmacy department, National Pirogov Memorial Medical University, Vinnytsya, Pyrogov str. 56, 21018 Vinnytsia, Ukraine; voronkina@vnmu.edu.ua
[2] Institute of Electronics and Sensor Materials, TU Bergakademie Freiberg, Gustav-Zeuner Str. 3, 09599 Freiberg, Germany; tsurkandd@gmail.com
[3] Faculty of Mechanical Engineering, Institute of Biomedical Engineering, Bialystok University of Technology, Wiejska Str. 45C, 15-351 Bialystok, Poland; e.romanczuk@pb.edu.pl (E.R.-R.); p.lipowicz@pb.edu.pl (P.L.)
[4] Center for Advanced Technology, Adam Mickiewicz University in Poznań, Uniwersytetu Poznańskiego 10, 61-614 Poznan, Poland;rprzekop@amu.edu.pl (R.E.P.); ewa.gabriel@amu.edu.pl (E.G.)
[5] Faculty of Chemistry, Adam Mickiewicz University in Poznań, 8 Uniwersytetu Poznańskiego, 61-614 Poznan, Poland; xmifrd@gmail.com
[6] Institute of Analytical Chemistry, TU Bergakademie Freiberg, Leipziger Str. 29, 09599 Freiberg, Germany; korbinian.heimler@chemie.tu-freiberg.de (K.H.); anika.rogoll@chemie.tu-freiberg.de (A.R.); carla.vogt@chemie.tu-freiberg.de (C.V.)
[7] Faculty of Physics, University of Warsaw, Pasteura 7, 02-093 Warsaw, Poland; pawel.wienclaw@sygnis.pl
[8] Department of Chemistry and Biochemistry, The University of Texas at Dallas, 800 W Campbell Rd, Richardson, TX 75080, USA; stelling@utdallas.edu
[9] International Institute of Biomineralogy GmbH, Am St.-Niclas Schacht 13, 09599 Freiberg, Germany;info@intib.eu
* Correspondence: herehr@amu.edu.pl

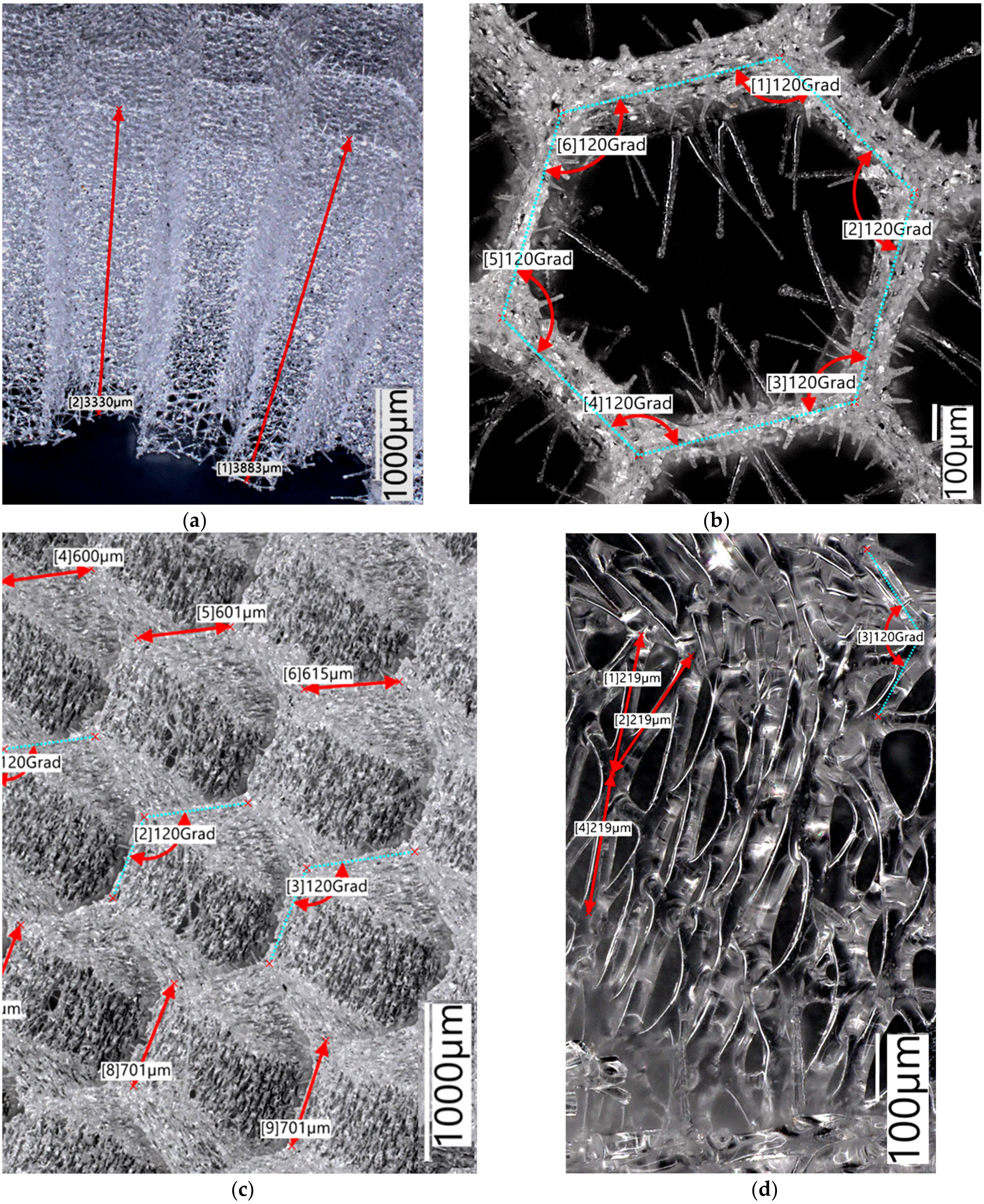

Figure S1. Digital microscopy imagery of selected fragments of purified siliceous skeleton of *A. beatrix* glass sponge. The skeleton wall is made of cylindrical honeycombs (**a**,**c**) with clear visible 120° angle motif (**b**,**c**). However, the walls of such cylinders possess triangular microarchitecture (side view, **d**). See also Figure S2**.**

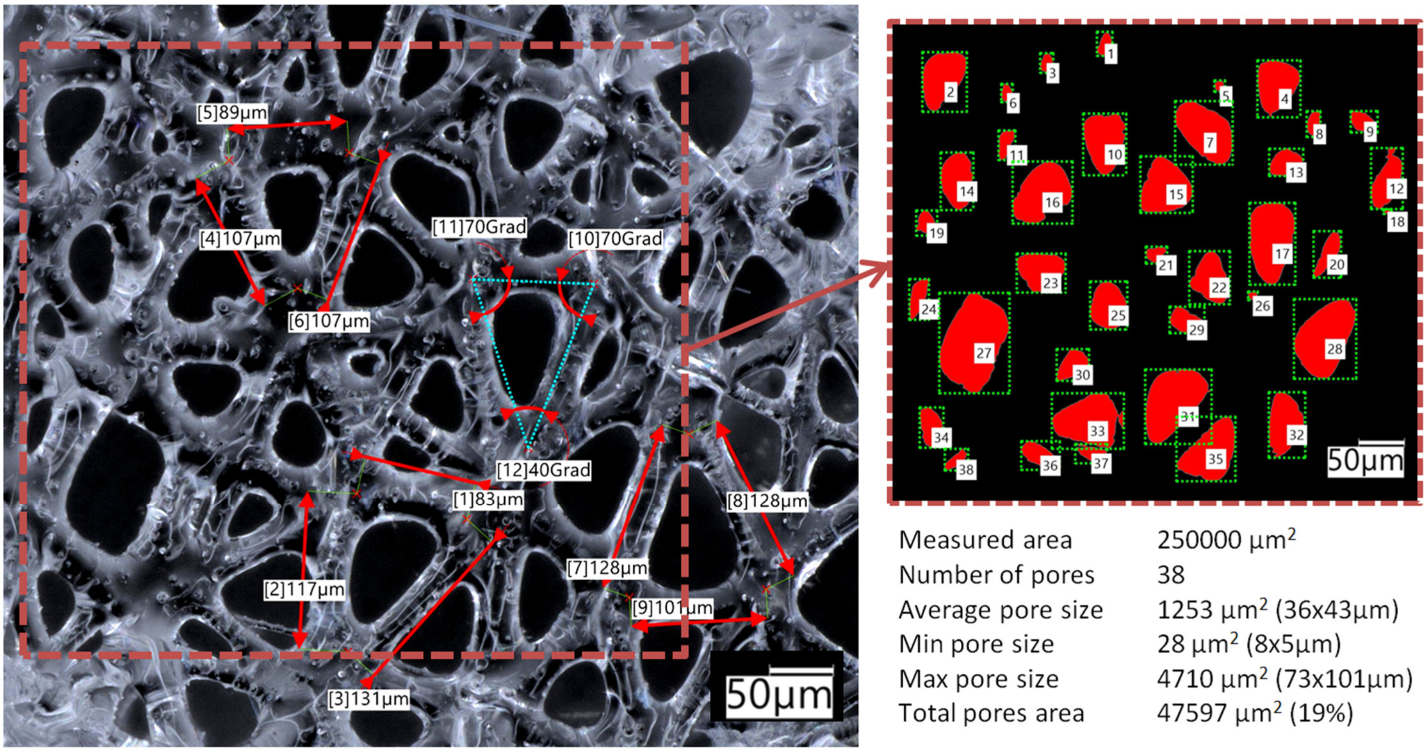

Figure S2. Digital microscopy image of the siliceous wall fragment (see **Figure S1, d**) that was selected for calculations of the pore size distribution. In contrast to regular, honeycomb-like structural motif of *A. beatrix* sponge skeleton (see **Figure S1 a-c**) in this case, there is both a large scatter in the pore sizes, and in the thickness, length and the angles of the glassy bridges. The calculations have been carried out using original software of Keyence VHX 7000 digital microscopy system.
